# Influence of *NQO1* Polymorphisms on Warfarin Maintenance Dose: A Systematic Review and Meta-Analysis (rs1800566 and rs10517)

**DOI:** 10.1155/2021/5534946

**Published:** 2021-08-12

**Authors:** Lihong Tian, Pingping Xiao, Bingrong Zhou, Yishan Chen, Lijuan Kang, Qingqing Wang, Jianfeng Lin, Min Son, Qingxiu Wu

**Affiliations:** ^1^Department of General Medicine, The Second Affiliated Hospital of Xiamen Medical College, Xiamen 361021, China; ^2^Department of Hematology/Rheumatology, The Second Affiliated Hospital of Xiamen Medical College, Xiamen 361021, China

## Abstract

This meta-analysis was conducted to analyze the effect of *NQO1* polymorphism on the warfarin maintenance dosage. Using strict inclusion and exclusion criteria, we searched PubMed, EMBASE, and the Cochrane Library for eligible studies published prior to July 7, 2021. The required data were extracted, and experts were consulted when necessary. Review Manager Version 5.4 software was used to analyze the relationship between *NQO1* polymorphisms and the warfarin maintenance dosage. Four articles involving 757 patients were included in the meta-analysis. Patients who were *NQO1* rs10517 G carriers (AG carriers or GG carriers) required a 48% higher warfarin maintenance dose than those who were AA carriers. Patients with *NQO1 rs1800566* CT carriers required a 13% higher warfarin dose than those who were CC carriers, with no associations observed with the other comparisons of the *NQO1 rs1800566* genotypes. However, the results obtained by comparing the *NQO1* rs1800566 genotypes require confirmation, as significant changes in the results were found in sensitivity analyses. Our meta-analysis suggests that the *NQO1* rs10517and *NQO1* rs1800566 variant statuses affect the required warfarin maintenance dose.

## 1. Introduction

Warfarin is an oral anticoagulant, widely used for the treatment and prevention of thromboembolic events owing to its anticoagulant efficacy and economic applicability, particularly in patients in developing countries. However, it has some disadvantages, such as a narrow therapeutic range, high interindividual variability in the maintenance dosage, and the risk of adverse effects, including the recurrence of thromboembolism and fatal bleeding risk [1].

Previous studies have shown that gene polymorphisms, including those in *CYP2C9*, *CYP4F2*, *VKORC1*, and *GGCX*, are associated with warfarin dosage adjustment [2–7]. In addition, clinical characteristics, such as height, body weight, and target International Normalized Ratio value, can influence the warfarin maintenance dosage [8–10]. However, these factors do not account for all current differences in warfarin dosage requirements among individuals and ethnicities [11]. Genetic polymorphism in addition to clinical factors can account for approximately 50% of the variability in the warfarin dosage requirements [12, 13], and *NQO1* genotypes may additionally explain the variance in the therapeutic warfarin dosage [14]. Moreover, a significant relationship between the *NQO1* (rs1800566 and rs15017) genotype and warfarin dosage has been observed [15, 16]; therefore, *NQO1* may contribute to warfarin dosage variation.

*NQO1* is located on chromosome 16 and encodes NAD(P)H dehydrogenase quinone 1, an enzyme that catalyzes the reduction of quinones, including vitamin K [14]. During vitamin K recycling, vitamin K is reduced to vitamin K hydroquinone by NQO1. Studies have reported that individuals carrying both mutated genomic alleles have no *NQO1* activity, whereas individuals heterozygous with one mutated allele have low-to-intermediate *NQO1* activity compared with wild-type individuals [17, 18]. Warfarin exerts its action by interfering with the cyclic interconversion of vitamin K to vitamin K epoxide [19]. Therefore, *NQO1* activity is associated with the enzyme's capacity to reduce vitamin K and the concentration of dicoumarol required to inhibit *NQO1* activity [20]. Since the discovery of *NQO1*, various *in vitro* and *in vivo* studies have reported that *NQO1* polymorphisms are important for maintaining vitamin K reduction in the absence of vitamin K epoxide reductase (VKOR) [21–23]. Previous studies showed that *NQO1* rs1800566 variants decrease the coagulation ability and ischemic stroke risk compared with the wild-type variant [24]. Therefore, *NQO1* rs1800566 polymorphisms likely influence the required dosage of warfarin. Momary et al. [25] focused on the effect of *NQO1* rs1800566 on warfarin dosage requirements. Subsequently, many studies of various patient populations have focused on the relationship between *NQO1* and warfarin dosage requirements and found a significant difference in the warfarin dosages among diverse *NQO1* genotypes [9, 15, 16, 25]. However, the role of diverse *NQO1* genotypes is controversial. Chung et al. reported that among Korean people, *NQO1* rs1800566 variant homozygote carriers required lower stable warfarin dosages than those with the wild-type allele [9]; El Rouby et al. indicated that *NQO1* rs1800566 variants were associated with higher dosage requirements among Latino populations [15]. No associations have been identified between *NQO1* rs1800566 and warfarin in populations of African or European ancestry [25, 26]. The role of *NQO1* genotypes may differ in people of different ethnicities. Although previous meta-analyses confirmed the effects of *CYP2C9*, *VORCK1*, *GGCX*, and *CYP4F2* genotypes on the warfarin dosage [2–6], studies of the influence of *NQO1* on warfarin dosage are limited. Therefore, we investigated the effects of individual *NQO1* genotypes on the mean daily warfarin dosage (MDWD) using meta-analyses.

## 2. Materials and Methods

### 2.1. Search Strategy

The search for eligible studies published in English prior to July 7, 2021, was conducted on PubMed, EMBASE, and the Cochrane Library. The search strategy used the following keywords: (*NQO1* OR “nicotinamide adenine dinucleotide phosphate, reduced quinone oxidoreductase”) AND (warfarin) AND (gene OR genotype OR genetics OR alleles OR polymorphism OR pharmacogenetics). The included literature references were examined, and the authors were contacted to obtain the necessary data.

### 2.2. Study Inclusion Criteria

The following stringent inclusion criteria were used: (1) all studies evaluated the relationship between warfarin maintenance dosage and *NQO1* polymorphism and (2) information on the warfarin maintenance dosage (mean and standard deviation, SD) and sample size for each *NQO1* genotype group was reported. It was not strictly required for patient characteristics (indication of warfarin, other interacting drugs, target International Normalized Ratio range, age, ethnicity, sex, etc.) to be reported in all studies.

### 2.3. Study Exclusion Criteria

Exclusion criteria included the following: (1) the publication types included review articles, abstracts, conference proceedings, and case reports; (2) unavailable data on mean warfarin dosage and SD and sample size for each *NQO1* genotype group; and (3) republished articles.

### 2.4. Data Extraction

Data were independently extracted, including clinical characteristics (such as name of the first author, publication year, sample size, target International Normalized Ratio range, indication of warfarin, predominant ethnicity, sex ratio, and mean age), sample size, allele frequencies, and warfarin maintenance dosage (mean and standard deviation, SD) for each *NQO1* genotype by two reviewers (TLH and ZBR). For inconsistent information, the two reviewers retrieved and discussed the original data to reach a consensus.

### 2.5. Study Quality Assessment

The quality assessment of the included studies was performed by two reviewers (TLH and XPP) independently. The Newcastle-Ottawa Scale was applied to assess the quality of all studies [27]. Each study was assigned a score of 0–9, and the studies were defined as high quality when the score was ≥7.

### 2.6. Statistical Analysis

In the meta-analysis, *NQO1* rs1800566 CT or TT carriers were defined as “rs1800566 T carriers,” whereas *NQO1* rs10517 AG or GG carriers were defined as “*NQO1* rs10517 G carriers.” Review Manager software version 5.4 was used to analyze the relationship between *NQO1* gene polymorphisms and the warfarin maintenance dosage. We used the inverse variance method to weight each study, and the mean difference (MD) was used to determine the effect of each *NQO1* genotype on the mean daily warfarin dosages (MDWD). To calculate the weighted mean difference (WMD), each MD was multiplied by the relative weight of each study. The sum of the WMD in each comparison was calculated to obtain the total WMD. In the meta-analysis, the *Z* test was applied to examine the influence of *NQO1* polymorphism on the warfarin maintenance dosage. The effect of the *NQO1* genotype was considered as significant when the *P* value was less than 0.05. The means of the Cochran's *Q* test (Mantel-Haenszel chi-squared test) was used to test heterogeneity in each genetic comparison group in the meta-analysis, and the results were expressed using the *P* value and *I*^2^ value. When the *P* value ≥ 0.1 or *I*^2^ value ≤ 25%, homogeneity was considered. A fixed effect model was also used to calculate the total WMD; otherwise, a random-effect model was selected.

To eliminate the source of heterogeneity, sensitivity analyses were performed by deselecting studies individually in a certain order. Moreover, Begg's test [28] and Egger's test [29] were applied to assess publication bias in each meta-analysis.

## 3. Results

### 3.1. Study Identification and Characteristics

**Figure 1:**
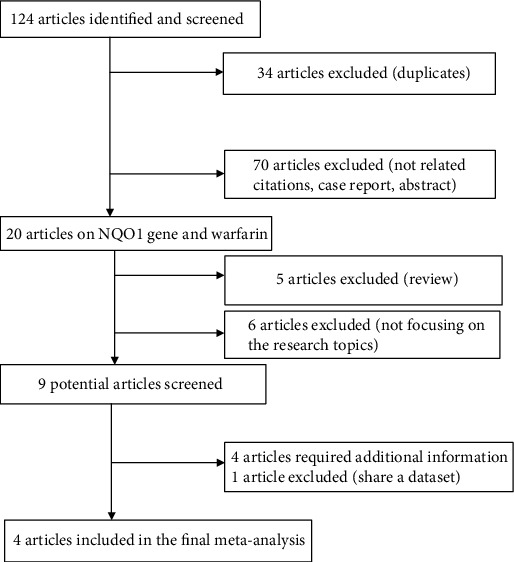
Flow diagram of the literature screening process used for the meta-analysis of *NQO1* polymorphisms and warfarin maintenance dosage.

A schematic of the literature screening process is shown in Figure 1. A total of 124 articles were screened, and four articles describing 757 patients were included in the meta-analysis [9, 25, 30, 31]. All included studies were published in English between 2007 and 2020. Only one article referred to African-Americans [25]. The other included studies referred to Asian populations [9, 30, 31]. Moreover, only two studies focused on the relationship between *NQO1* rs10517 genotypes and MDWDs [9, 30]. According to the Newcastle-Ottawa Scale checklist, all studies with scores of 7 stars were considered as high quality. The characteristics of the four studies used in the meta-analysis are shown in Table 1.

### 3.2. Meta-Analysis

#### 3.2.1. Relationship between NQO1 rs1800566 and Warfarin Maintenance Dosage

**Figure 2:**
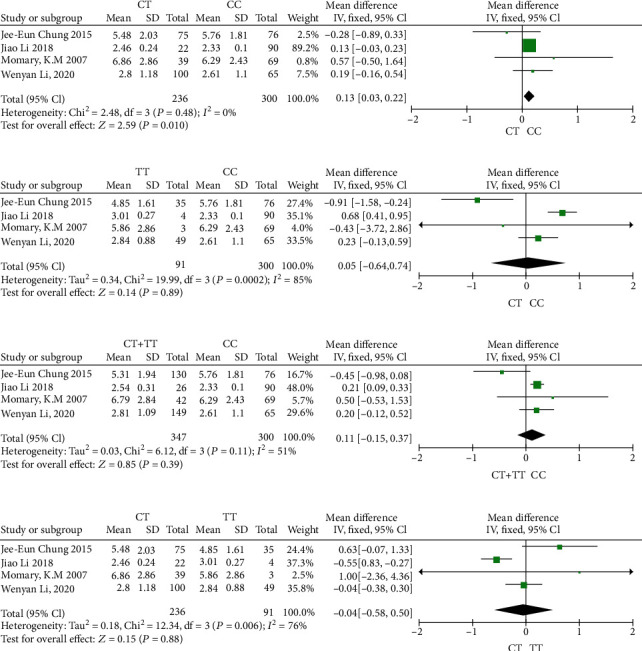
Forest plots illustrating the influence of *NQO1* rs1800566 polymorphisms on warfarin dose requirement. (a) Relative warfarin dosage requirement of rs1800566 CT carriers compared to CC carriers (wild-type). (b) Relative warfarin dosage requirement of rs1800566 TT carriers versus CC carriers. (c) Relative warfarin dosage requirement of rs1800566 T carriers (CT or TT carriers) versus CC carriers. (d) Relative warfarin dosage requirement of rs1800566 CT carriers versus TT carriers. Mean (SD): mean and standard deviation of normalized warfarin doses associated with each genotype. IV: inverse variance; CI: confidence interval. Values in the square brackets denote 95% confidence intervals.

There were four articles [9, 25, 30, 31] included in this meta-analysis, and the results are shown in Figure 2. Compared with the *NQO1 rs1800566* homozygous genotype (CC carriers), the warfarin dose in patients with the CT genotype was 13% higher (*P* value = 0.01, 95% confidence interval (CI) 0.03–0.22). Analysis using a fixed-effect model revealed homogeneity. No significant difference was found in the other comparisons (TT versus CC: *P* = 0.89; T carriers versus CC: *P* = 0.39; CT versus TT: *P* = 0.88), and a random-effect model was applied in the other comparison of *NQO1 rs1800566* genotype groups as significant heterogeneity was detected (all *P* values < 0.1, *I*^2^ value > 50%).

#### 3.2.2. Relationship between NQO1 rs10517 and Warfarin Maintenance Dosage

**Figure 3:**
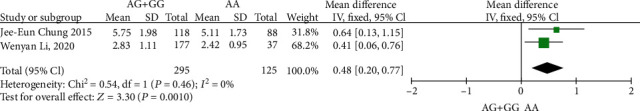
Forest plots showing the influence of *NQO1* rs10517 on warfarin maintenance dosage. Relative warfarin dosage requirement of *NQO1* rs10517 G carriers (GA or GG carriers) compared to AA carriers (wild-type). Mean (SD): mean and standard deviation of normalized warfarin doses associated with each genotype. WMD: weighted mean differences; CI: confidence interval.

Only two studies [9, 30] were included in this meta-analysis, which included 420 patients. Because of the limited number of patients with each genotype, we only conducted comparisons between *NQO1* rs10517 G carriers and AA carriers. The results are shown in Figure 3. Compared with the AA carriers, G carriers required significantly higher MDWD values (*P* = 0.001). Homogeneity was observed in the meta-analysis (*P* = 0.46, *I*^2^ = 0%), and thus, a fixed-effect model was used.

### 3.3. Sensitivity Analysis

Sensitivity analyses were performed by individually deselecting studies. No significant change was found in the comparison between *NQO1* rs10517 G carriers and AA carriers, whereas a significant change was found in comparisons of *NQO1* rs1800566 (CT versus CC; TT versus CC; T carriers versus CC). Researchers have suggested that *NQO1* significantly influences the warfarin maintenance dosage in some Asian populations [15]; therefore, we removed one study that was not conducted in Asian populations [25] and performed a meta-analysis on *NQO1* rs1800566 and warfarin maintenance dosage in Asian populations. There was no difference between Asian and all populations (data shown in Table 2).

### 3.4. Publication bias

Begg's and Egger's tests were applied in each meta-analysis. No publication bias was found in the association between *NQO1* polymorphisms and warfarin dosage (data shown in Table 3).

## 4. Discussion

*NQO1* is thought to be one of the gene polymorphisms influencing warfarin maintenance dosage [14–16]. In the vitamin K cycle, vitamin K is reduced to vitamin K hydroquinone by NAD(P)H:quinone oxidoreductase 1 (NQO1). The ability of NQO1enzyme activities was affected by the *NQO1* genotype, especially *NQO1* rs1800566 [32]. When a C was replaced by T at position 609 of the cDNA, the proline was replaced by the serine at amino acid 187 of the functional protein, leading to a possible loss of external interactions with other moieties necessary for its proper functioning [32]. It was proven that individuals with the *NQO1* rs1800566 variant homozygote have no *NQO1* activity, and individuals who are *NQO1* rs180056 heterozygous have low-to-intermediate *NQO1* activity compared to wild-type ones [18, 33]. Warfarin exerts its action by interfering with the conversion of vitamin K epoxide to vitamin K in the vitamin K cycle, leading to reduced activation of vitamin K-dependent clotting factors [19]. Therefore, *NQO1* probably affects warfarin maintain dosage.

Previous meta-analyses on the role of *CYP2C9*, *VKORC1*, and *CYP4F2* polymorphisms in the warfarin maintenance dosage have been conducted [4, 6, 34, 35]. However, these studies did not completely explain the variation in therapeutic warfarin dosages [14, 16]. *NQO1* genotypes could be one factor which could affect warfarin dosage [21, 23], whereas the relationship between *NQO1* genotypes and warfarin maintenance dosage was controversial. In 2012, Bress et al. confirmed that *NQO1* rs1800566 genotypes significantly affected the warfarin-required dosage and predicted that patients with variant genotypes required higher warfarin dosages [14]. Chung et al. showed that patients with *NQO1* rs1800566 variant genotypes required lower warfarin maintenance dosages; patients with *NQO1* rs10517 variant genotypes required higher warfarin maintenance dose compared with those who were wild-type homozygotes [9]. However, some studies found no significant association between *NQO1* polymorphism and the warfarin maintenance dosage requirements [30, 31]. Although Asiimwe et al. verified that *NQO1* rs1800566 has no significant influence on warfarin dosage in Black-African patients by meta-analysis, the role of *NQO1* genotypes in warfarin maintenance in other ethnic groups was not investigated, the relationship between other *NQO1* genotypes and warfarin dosages was not researched, and the study sample size was small [36]. Until now, the relationship between *NQO1* genotypes and warfarin dosage remains unclear, especially in non-Black-African patients. Therefore, we conducted a meta-analysis to analyze the relationship between *NQO1*polymorphisms (rs1800566 and rs10517) and the warfarin maintenance dosage.

We found that *NQO1* rs10517 G carriers required 48% higher MDWD than AA carriers. In patients with *NQO1 rs1800566*, those with the CT genotype required 13% higher MDWD than those with the CC genotype, which was significant, whereas no significant difference was found in the other comparisons (TT versus CC; T carriers versus CC; CT versus TT).

In our meta-analysis, we selected two single nucleotide polymorphisms (SNPs) of the *NQO1* gene, rs1800566 and rs10517, which have proven to be significantly associated with warfarin maintenance doses. However, our results showed that the effects of two SNPs on warfarin maintenance dosages were inconsistent. This may be due to the differing mechanism of the two SNPs on warfarin. Rs10517 in the 3′UTR region of the *NQO1* gene might alter the function of the protein by affecting the secondary structure of mRNA and upregulating the coagulation factor activity [37], whereas *NQO1* rs1800566 affects vitamin K metabolism and alters warfarin response [9].

Sensitivity analyses performed by deselecting studies individually in a certain order revealed no significant change in the comparison between *NQO1* rs10517 G carriers and AA carriers, suggesting that our results were stable and reliable. Significant changes were found in the comparisons of the *NQO1* rs1800566 genotype (CT versus CC; TT versus CC; T carriers versus CC). Therefore, the results of the *NQO1* rs1800566 genotype comparisons must be considered with caution.

It is known that the frequency of *VKORC1* and *CYP2C9* significantly differs in different ethnic groups, and the contributions of the *VKORC1* and *CYP2C9* genotypes to warfarin dose requirements are inconsistent in different ethnic populations [15]. Additionally, it is thought that *NQO1* influences the warfarin maintenance dosage significantly in some Asian populations [15]; therefore, we removed the study in African-Americans [25] and conducted a meta-analysis of *NQO1* rs1800566 and the warfarin maintenance dosage in Asian populations. However, compared with all populations, the results showed no significant difference, possibly because of the limited number of articles and sample size of *NQO1* rs1800566. No Caucasian population was included in the meta-analysis. Thus, the influence of *NQO1* genotypes on the warfarin dosage in Caucasians is unclear in our meta-analysis.

Our meta-analysis showed that patients with the *NQO1* rs1800566 CT genotype required a higher warfarin dosage than those with the CC genotype (wild-type genotype), and the level of evidence of *NQO1* rs1800566 for warfarin dosage is grade 3(available at http://www.pharmgkb.org/). However, the result was in contrast to the findings of previous studies [17, 24]. This may be because the *NQO1* variant allele was likely in linkage disequilibrium with the functional single-nucleotide polymorphism in some populations [15]. Therefore, studies are required to focus on the underlying reason for the *NQO1* association. In addition, no significant difference was found in the other comparisons of *NQO1* rs1800566 (TT versus CC; T carriers versus CC; CT versus TT), possibly because the number of included studies and sample size of patients with the *NQO1* rs1800566 genotype was small. Although patients with the *NQO1* rs10517 variant genotypes (AG carriers and GG carriers) required higher warfarin maintenance doses than those with the wild-type homozygote (AA carriers) genotype in our meta-analysis, the effect of the *NQO1* rs10517 genotype on warfarin should be considered with caution because the number of included studies was limited. Further analysis of the relationship between *NQO1* genotypes (rs1800566 and rs10517) and the warfarin maintenance dosage is required to confirm our results.

Our study had some limitations. First, the number of articles used was small, including only four studies on *NQO1* rs1800566 and two studies on *NQO1* rs10517. Second, the studies only included patients of African-American and Asian descent. Third, some studies showed a significant effect between *NQO1*genotypes, and the warfarin dosages were not included because of lack of data. Further studies are needed to support our findings.

## 5. Conclusions

This meta-analysis focused on the relationship between the warfarin maintenance dosage and *NQO1* polymorphism. We found that *NQO1 rs1800566* CT carriers required a 13% higher warfarin dose compared to CC carriers, and no associations were observed in the other comparisons of *NQO1 rs1800566* genotypes. *NQO1* rs10517 G carriers required a 48% higher warfarin dose than AA carriers. *NQO1 rs1800566* polymorphisms and *NQO1* rs10517 polymorphisms may significantly affect individual warfarin dosage requirements. Additional studies of larger sample sizes in multiethnic populations are required to avoid selection bias and confirm our findings.

## Figures and Tables

**Table 1:** Basic characteristics and quality score results of the included study in the systematic review.

Study	Number (men %)	Predominant ethnicity	Indication of warfarin	Target INR range	HWE	Mean ages (years)	Number/frequency (%)			Dose (mg/day)			Quality
							CC	CT	TT	CC	CT	TT	
rs1800566													
Momary et al. (2007)	115 (28)	African-Americans	∗	2.5-3.5 (2.5, 94%)	Yes	57 ± 16	69/62	39/35	3/3	6.29 ± 2.43	6.86 ± 2.86	5.86 ± 2.86	High
Chung et al. (2015)	206 (32.5)	Korean	MCA	2.0-3.0	Yes	61.2 ± 10.2	171/83		35/17	5.61 ± 1.94		4.85 ± 1.61	High
Li et al. (2018)	222 (36.9)	Chinese	AF	#	Yes	47.5 ± 0.72	90/78	22/19	4/3	2.33 ± 0.1	2.46 ± 0.24	3.01 ± 0.27	High
Li et al. (2020)	214 (52.8)	Chinese	AF, PE, DVT, other	1.6–2.8	Yes	72.6 ± 11.2	65/30.4	100/46.7	49/22.9	2.61 ± 1.10	2.80 ± 1.18	2.84 ± 0.88	High

rs10517							AA	AG	GG	AA	AG	GG	Quality
Chung et al. (2015)	260 (74)	Korean	MCA	2.0-3.0	Yes	61.2 ± 10.2	88/42.7	118/57.3		5.11 ± 1.73	5.75 ± 1.98		High
Li et al. (2020)	214 (52.8)	Chinese	AF, PE, DVT, other	1.6–2.8	Yes	72.6 ± 11.2	37/17.3	101/47.2	76/35.5	2.42 ± 0.95	2.87 ± 1.25	2.77 ± 0.88	High

VT: venous thromboembolism; DVT: deep vein thrombosis; AF: atrial fibrillation; MCA: mechanical cardiac valves; PE: pulmonary embolism; IS: ischemic stroke; CAD: coronary artery disease. ^∗^Hypertension, VT, IS/TIA, diabetes, CAD, etc.; ^#^target INR range = 1.5–2.0 (54.1%), 2.0–2.5 (37.4%), 2.5–3.0 (8.5%).

**Table 2:** The mean values of warfarin dose for each genotype and comparison of the effect of variant *NQO1* rs1800566 genotypes on warfarin dosage requirement in Asian and all populations.

Groups	All population		*P* value	Asian population		*P* value
	MDWD	WMD (95% CI)		MDWD	WMD	
CC	4.17 ± 2.38	—	—	3.54 ± 1.96	—	—
CT	4.29 ± 2.53	—	—	3.78 ± 2.12	—	—
TT	3.72 ± 1.63	—	—	3.65 ± 1.55	—	—
CT vs. CC	—	13% (3, 22%)	0.01	—	12% (3, 22%)	0.01
TT vs. CC	—	5% (-64%, 74%)	0.89	—	7% (-66%, 79%)	0.86
CT vs. TT	—	-4% (-58%, 50%)	0.88	—	-6% (-63%, 50%)	0.82
T carriers vs. CC	—	11% (-15%, 37%)	0.39	—	8% (-21%, 37%)	0.60

MDWD: mean daily warfarin dosages; WMD: weighted mean differences; CI: confidence interval; the inverse variance method was used in all comparisons of weight in each study.

**Table 3:** Begg's test for each meta-analysis.

SNP		Begg's test
rs10517	G carriers versus AA	Pr > ∣*z* | = 1.000

rs1800566	CT versus CC	Pr > ∣*z* | = 0.734
	TT versus CC	Pr > ∣*z* | = 0.734
	CT versus TT	Pr > ∣*z* | = 0.734
	T carriers versus CC	Pr > ∣*z* | = 0.734
